# Effects of perforation size on the success rate of tympanoplasty using a cartilage graft^☆^

**DOI:** 10.1016/j.bjorl.2016.10.009

**Published:** 2016-11-17

**Authors:** Zhufang Jiang, Zihan Lou

**Affiliations:** a Yiwu Central Hospital, Department of Otorhinolaryngology, Yiwu City, Zhejiang Province, China; b Xinxiang Medical University, Department of Clinical Medicine, Henan, China

Dear Editors,

Here, we review the article entitled “Impact of cartilage graft size on success of tympanoplasty” by Abdelhameed et al.^1^ The authors evaluated the effects of different perforation sizes on the outcomes of Type 1 tympanoplasty in this study. The work described is interesting. However, the inclusion criteria and the results are not very clear. The authors do not state whether patients with myringosclerosis were included in the methods section. Myringosclerosis affects the blood supply to, and nutrition of, the eardrum, causing graft failure after Type 1 tympanoplasty. Migirov et al.^2^ believed that appropriate freshening of the perforation edges, with removal of sclerotic plaques, improved the success rate of tympanoplasty in patients with myringosclerosis.

The study divided 50 patients into three groups. Group I had perforations of more than 50% of the Tympanic Membrane (TM); Group II had perforations of between 25% and 50% of the TM; and Group III had perforations ≤25% of the TM. The three groups contained 16, 20, and 14 patients, respectively.^1^ The authors write in the Results: “Fifty patients underwent cartilage tympanoplasty, thirty seven patients had unilateral perforation, while 13 had bilateral perforations. The anatomical success rate defined as graft take after 12 months of follow up among all patients was 92%, 4 patients underwent revision surgery 10–12 months postoperatively. Two of them were in first group, and one patient in each remaining groups, no statistical difference was noted among the three groups regarding failure percentage”.^1^ The authors state only that 50 patients underwent cartilage tympanoplasty, but do not explain in detail how many perforations were treated. If the success rate is based on patient numbers, the success rates were 87.5% (14/16) in Group I, 95% (19/20) in Group II, and 92.9% (13/14) in Group III. Although no significant differences were evident among the three groups in terms of failure percentage, the success rate in Group II was 10% higher than that in Group I. The sample numbers of the three groups were very small; we believe that the differences between Group II and the other groups would have been significant if the sample numbers been larger. Previous studies have suggested that the preoperative size of the perforation may affect the success rate of Type 1 tympanoplasty using a cartilage graft.^3^, ^4^ Wu et al.^5^ compared the short- and long-term hearing outcomes of patients with small and large eardrum perforations who underwent successful inlay cartilage tympanoplasty. No differences were apparent between the short- and long-term air–bone gap closure (*p* = 0.689) of small perforations; however, a significant difference between short- and long-term closure (*p* = 0.029) was evident in patients with large perforations. Thus, large sample sizes of patients with perforations of different size, and long-term follow-up, are needed in future studies.

## Conflicts of interest

The authors declare no conflicts of interest.
